# Identification of methylation signatures and rules for predicting the severity of SARS-CoV-2 infection with machine learning methods

**DOI:** 10.3389/fmicb.2022.1007295

**Published:** 2022-09-23

**Authors:** Zhiyang Liu, Mei Meng, ShiJian Ding, XiaoChao Zhou, KaiYan Feng, Tao Huang, Yu-Dong Cai

**Affiliations:** ^1^School of Life Sciences, Changchun Sci-Tech University, Changchun, China; ^2^State Key Laboratory of Oncogenes and Related Genes, Center for Single-Cell Omics, School of Public Health, Shanghai Jiao Tong University School of Medicine, Shanghai, China; ^3^School of Life Sciences, Shanghai University, Shanghai, China; ^4^Department of Computer Science, Guangdong AIB Polytechnic College, Guangzhou, China; ^5^Bio-Med Big Data Center, CAS Key Laboratory of Computational Biology, Shanghai Institute of Nutrition and Health, University of Chinese Academy of Sciences, Chinese Academy of Sciences, Shanghai, China; ^6^CAS Key Laboratory of Tissue Microenvironment and Tumor, Shanghai Institute of Nutrition and Health, University of Chinese Academy of Sciences, Chinese Academy of Sciences, Shanghai, China

**Keywords:** SARS-CoV-2, severity, methylation, machine learning, classification rule

## Abstract

Patients infected with SARS-CoV-2 at various severities have different clinical manifestations and treatments. Mild or moderate patients usually recover with conventional medical treatment, but severe patients require prompt professional treatment. Thus, stratifying infected patients for targeted treatment is meaningful. A computational workflow was designed in this study to identify key blood methylation features and rules that can distinguish the severity of SARS-CoV-2 infection. First, the methylation features in the expression profile were deeply analyzed by a Monte Carlo feature selection method. A feature list was generated. Next, this ranked feature list was fed into the incremental feature selection method to determine the optimal features for different classification algorithms, thereby further building optimal classifiers. These selected key features were analyzed by functional enrichment to detect their biofunctional information. Furthermore, a set of rules were set up by a white-box algorithm, decision tree, to uncover different methylation patterns on various severity of SARS-CoV-2 infection. Some genes (PARP9, MX1, IRF7), corresponding to essential methylation sites, and rules were validated by published academic literature. Overall, this study contributes to revealing potential expression features and provides a reference for patient stratification. The physicians can prioritize and allocate health and medical resources for COVID-19 patients based on their predicted severe clinical outcomes.

## Introduction

Since its outbreak in late 2019, COVID-19, which is caused by SARS-CoV-2, has resulted in more than 5 million deaths. SARS-CoV-2 binds to the spike (S) protein primarily through its functional receptor ACE2, an 805-amino acid type I transmembrane protein, allowing the virus to attach to the host cell membrane. This process results in alteration of the extracellular domain of ACE2 and internalization of the transmembrane domain, leading to further fusion of the viral particle with the host cell (Schulte-Schrepping et al., 2020). The SARS-CoV-2 infection progresses to different severities, including discharge from the emergency department, hospitalization, transfer to the ICU, and death, due to a variety of factors, such as age, gender, and other underlying diseases (Konigsberg et al., 2021). Therefore, rapidly determining the severity of the patient and taking corresponding treatment measures for timely and effective diagnosis and treatment is crucial.

Viruses can escape the immune clearance of the body through a variety of ways, among which epigenetic modification is an important way for respiratory viruses to resist the immune response of the body. DNA methylation, mainly of CpG islands, is a crucial reversible epigenetic regulation process (Fan et al., 2017; Benhamida et al., 2020). The regulation of the activity of a variety of DNA/RNA viruses, including HIV, HBV, and HPV, is related to changes in DNA methylation (Castro De Moura et al., 2021). Studies have shown that MERS-CoV and H5N1 influenza virus infection leads to methylation of antigen-presenting gene promoters in infected cells, which eliminates the expression of related genes, thereby antagonizing antigen presentation, resulting in impaired T-lymphocyte function during acute infection and aggravating the degree of virus infection in the body (Hatta et al., 2010; Menachery et al., 2018). Similarly, as a respiratory virus, SARS-CoV infection also results in DNA methylation in host cells (Menachery et al., 2018). Among them, the hypermethylation of the IFN pathway and inflammation-related genes is an important feature of severe COVID-19 (Corley et al., 2021). The study of ACE2 revealed that the DNA in the CpG island of the ACE2 promoter in lung epithelial cells is hypomethylated, indicating its high expression in the lung. Moreover, its methylation status was significantly correlated with age and gender, explaining the effect of age and gender on the severity of COVID-19 (Kianmehr et al., 2021). In addition, ACE2 mRNA is highly expressed in various diseases, especially cancer, which may be an important reason for the severe COVID-19 caused by the underlying disease in SARS-CoV infection (Sen et al., 2021). RNA modification, namely N 6-methylation of adenosine (m6A), also plays an important role in evading the innate immune recognition of exogenous RNA of the host, affecting virus structure and replication (Eberle et al., 2021). The study of human metapneumovirus showed that m6A-binding protein can label viral RNA as the RNA of the host after binding to m6A, thereby evading the antiviral response of the host (Durbin et al., 2016; Chen et al., 2019). In addition, some studies have found that the N region of the SARS-CoV-2 virus genome is rich in m6A modification and is regulated by the host cell methyltransferase METTL3. The reduced expression level of METTL3 will lead to a decrease in the level of SARS-CoV-2 m6A and correspondingly increased expression of inflammatory genes (Li S. et al., 2021). This process is more pronounced in severely infected patients than that in moderately infected patients. These findings suggest the possibility of using methylation to characterize disease states, and numerous studies have demonstrated the feasibility of this approach.

This study conducted a computational investigation on the blood methylation profile on severity of SARS-CoV-2 infection. Several advanced machine learning methods were adopted. First, the profile was analyzed by the Monte Carlo feature selection (MCFS) method (Dramiński et al., 2007) to analyze the importance of methylation features. One feature list was produced, which was further analyzed by incremental feature selection (IFS) (Liu and Setiono, 1998) method. Four classification algorithms were adopted in the IFS method to discover their optimal features, and build the optimal classifiers and classification rules. For the essential methylation features, their corresponding genes were picked up for gene ontology (GO) and KEGG enrichment analysis. Some results, including essential methylation sites, classification rules, and enrichment analysis results, were extensively discussed and can be validated by existing literature. The results reported in this study are helpful for the stratification of clinical patients and provide an effective reference for clinical diagnosis and treatment.

## Materials and methods

### Methylation dataset

The blood DNA methylation dataset investigated in this study was obtained from the Gene Expression Omnibus (GEO) database with the accession ID of GSE167202 (Konigsberg et al., 2021). This dataset comprised 164 SARS-CoV-2-positive samples, 296 SARS-CoV-2-negative infection samples, and 65 other infection samples. In addition, the positive samples were classified into four categories based on severity score. The severity score is determined primarily by discharge from emergency, admission to inpatient care, progression to the ICU, and death. The above four categories, negative infection, and other infections were termed as six classes in this study. The methylation dataset was deeply analyzed by modeling a classification problem on the dataset. The sample size of each class is listed in Table 1. Each sample was represented by 655,010 methylation features. This dataset would be analyzed in the following steps.

**Table 1:** Sample size of each class for the methylation profile.

Class name	Sample size
Negative infection	296
Other infection	65
Discharged from emergency department	34
Admitted to inpatient care	84
Progressed to ICU	35
Death	11

### Monte Carlo feature selection

A large number of methylation features were used to represent each sample. However, only a few of them were highly related to the severity of SARS-CoV-2 infection. It was necessary to reveal essential methylation features with advanced computer techniques. Here, MCFS method was employed (Dramiński et al., 2007).

MCFS is a tree-based feature selection method that is widely used in methylation profiling analysis as it is deemed to be good at dealing with datasets containing small number of samples and huge number of features. It randomly constructs several decision trees (DTs) from the original training dataset and uses these DTs to evaluate the importance of features. More specifically, *s* subsets with *m* features are randomly selected from the original training dataset. *t* trees for each subset are then constructed based on samples randomly sampled from the original dataset. The performance of each tree is evaluated on test samples that are not selected as training samples. Overall, s×t DTs are built in this process. The overall position of a feature on the tree node partition is used to estimate a measurement, called relative importance (RI). A high RI score of a feature indicates the importance of a feature. The RI score is defined as follows:


(1) $$RI=\sum_{\tau =1}^{st}{\left(wACC\right)}^{u}\sum_{n_{g}\left(\tau \right)}^{}IG\left(n_{g}\left(\tau \right)\right){\left(\frac{no.in\;n_{g}\left(\tau \right)}{no.in\;\tau }\right)}^{v},$$

where $IG\left(n_{g}\left(\tau \right)\right)$ indicates the information gain of tree node $n_{g}\left(\tau \right)$, $no.in\;n_{g}\left(\tau \right)$ and no.inτ represent the number of samples in node $n_{g}\left(\tau \right)$ and tree τ, respectively, and wAcc indicates the weighted accuracy of the DT τ. In addition, *u* and *v* are the two parameters for RI calculation.

The MCFS program developed by Dramiński et al. was applied in this study, which can be downloaded at https://home.ipipan.waw.pl/m.draminski/mcfs.html, to rank the methylation features. Default parameters were used, where *u* and *v* were set to 1. By applying the MCFS program on the methylation dataset, a ranked feature list was obtained.

### Incremental feature selection

Based on the MCFS method, the methylation features were ranked in a list. However, the threshold was difficult to determine, that is, which features were selected for further analysis. In view of this, we further employed the IFS method (Liu and Setiono, 1998).

The IFS method is always used to determine the optimal number of features in a ranked feature list combined with one supervised classification algorithm, such as random forest (RF). More specifically, IFS first generates a series of feature subsets based on a step size. For example, the first and second subsets, respectively, comprise the top 5 and 10 features when the step size is five. Next, on each feature subset, the samples represented by features in such subset are learned by the given classification algorithm, thereby building a classifier. Its performance is evaluated by the 10-fold cross-validation (Kohavi, 1995). After the evaluation metrics of all classifiers are obtained, the classifier with the highest performance is easy to find. Such classifier is called the optimal classifier. The corresponding feature subset is picked up and features in this subset are termed as the optimal features for the used classification algorithm.

### Synthetic minority oversampling technique

As shown in Table 1, the sample sizes under six classes were quite different. The largest class contained samples about 17 times as many as those in the smallest class. This may lead to biased performance of the established classifiers. Therefore, the synthetic minority oversampling technique (SMOTE) algorithm (Chawla et al., 2002; Ding et al., 2022; Zhou et al., 2022), an oversampling method, was applied to solve the problem. The core idea of SMOTE is to generate new samples to each minor class for enlarging its size. For each minor class, SMOTE randomly selects one sample, say *x*, from this class and finds its k-nearest neighbor samples in the same class. One sample, say *y*, is randomly selected from these k-nearest neighbor samples. One new sample is synthesized by the linear combination of *x* and *y*. As such new sample is highly related to *x* and *y*, it belongs to the same class with a high probability. Thus, it is put into the minor class. Such procedures execute several times until the size of the minor class is equal to that of the major class.

In this study, the SMOTE program from the imblearn package^1^ was used to process the methylation data for solving the imbalanced problem when constructing classifiers in the IFS method.

### Classification algorithm

As the execution of IFS method needs one classification algorithm, four classic classification algorithms were attempted in this study to fully assess each constructed feature subset. They were k-nearest neighbor (kNN; Cover and Hart, 1967), RF (Breiman, 2001), support vector machine (SVM; Cortes and Vapnik, 1995), and DT (Safavian and Landgrebe, 1991). Their brief descriptions were as follows.

#### k-Nearest neighbor

k-Nearest neighbor is one of the most classic classification algorithms. It determines the class of a sample based on measuring the distance between samples. Given a training dataset, for a new test sample, the *k* neighbors closest to such sample are found in the training dataset. By counting the classes of its *k* neighbors, the class of the test sample can be determined. Generally, the class that occurs most for its *k* neighbors is assigned to the test sample.

#### Random forest

RF is an ensemble algorithm that contains several DTs. Each DT is constructed by randomly selecting samples from the original dataset and features from all features. RF provides the final prediction result using the voting strategy on predictions yielded by DTs. RF is generally much more powerful than its component DT, and few parameters are involved in this algorithm.

#### Support vector machine,

SVM is an excellent classification algorithm in machine learning. The original SVM can only tackle binary classification. It separates samples into two classes by constructing a hyperplane, which can separate samples into two classes with the maximum interval. However, such hyperplane does not always exist or is not easy to find out. SVM maps samples into a high-dimensional space using one kernel function. In the new space, the hyperplane can be easily constructed. For a test sample, it is also mapped into the high-dimensional space. Its class is determined by the side it lies. The “one-versus-rest” or “one-versus-one” can be adopted to generalize the original SVM so that it can tackle multi-class classification problems.

#### Decision tree

Different from the above algorithms, which are deemed as black-box algorithms, DT can make the classification procedures interpretable. By learning the distributions of samples under each feature, a tree-like structure is built by DT. In this structure, each internal node indicates a decision on an attribute, outputting a judgment result, and each leaf node denotes a classification outcome. Besides, DT can also be represented by a set of rules. Each rule is obtained by a path from the root node to one leaf node in the tree. In terms of these rules, the class of a test sample can be determined. This operation also makes the classification procedures completely open, giving more chances for us to understand the procedures. In this case, more meaningful and hidden information in the dataset can be mined.

Above classification algorithms have wide applications in many fields. They are always important candidates for building classifiers in tackling various biological and medical problems (Zhou et al., 2020a,b, 2022; Chen et al., 2021, 2022; Onesime et al., 2021; Zhang Y. et al., 2021; Ding et al., 2022; Li et al., 2022; Ran et al., 2022; Tang and Chen, 2022; Wang and Chen, 2022; Wu and Chen, 2022; Yang and Chen, 2022). These algorithms were implemented in this study through the scikit-learn (Pedregosa et al., 2011) program in Python and run with default parameters.

### Performance measurement

The prediction performance of each classifier was mainly evaluated with the weighted F1. Its calculation is based on the F1 score on each class. The F1 score for one class can be computed by


(2) $$\mathrm{Precision}=\frac{TP}{TP+FP},$$

(3) $$\mathrm{Recall}=\frac{TP}{TP+FN},$$

(4) $$F1\;\mathrm{score}=2\times \frac{\mathrm{precision}\times \mathrm{recall}}{\mathrm{precision}+\mathrm{recall}},$$

where *TP*, *FP*, and *FN* represent true-positive, false-positive, and false-negative for the class, respectively. The weighted F1 is defined as the weighed mean of F1 scores on all classes. The direct mean of F1 scores on all classes was also provided, which was called macro F1.

To fully evaluate the performance of classifiers in the IFS method, we also adopted overall accuracy (ACC) and Matthews correlation coefficients (MCC; Matthews, 1975; Gorodkin, 2004). ACC is defined as the ratio of correctly predicted samples and all samples, which is the most accepted measurement. However, it is not perfect when the class sizes are of great differences. In view of this, MCC was proposed, which is deemed as a balanced measurement. For computing MCC, two binary matrices *X* and *Y* should be constructed first, where *X* stands for the true class of each sample and *Y* represents the predicted class of each sample. Then, MCC can be computed by


(5) $$\mathrm{MCC}=\frac{\mathrm{cov}\;\left(\mathrm{X,Y}\right)}{\sqrt{\mathrm{cov}\;\left(\mathrm{X,X}\right)\mathrm{cov}\left(\mathrm{Y,Y}\right)}}$$

### Enrichment analysis

According to the IFS results, the essential methylation features for severity of SARS-CoV-2 infection can be obtained. Their corresponding genes can be picked up for further analysis. GO and KEGG enrichment analysis is a common method for uncovering biological meanings behind a set of genes. Here, it was applied to discover the biofunctional information of the genes corresponding to essential methylation features. Such analysis was performed by using the R package clusterProfiler 4.0 (Wu et al., 2021) with a threshold of 0.05.

## Results

**Figure 1:**
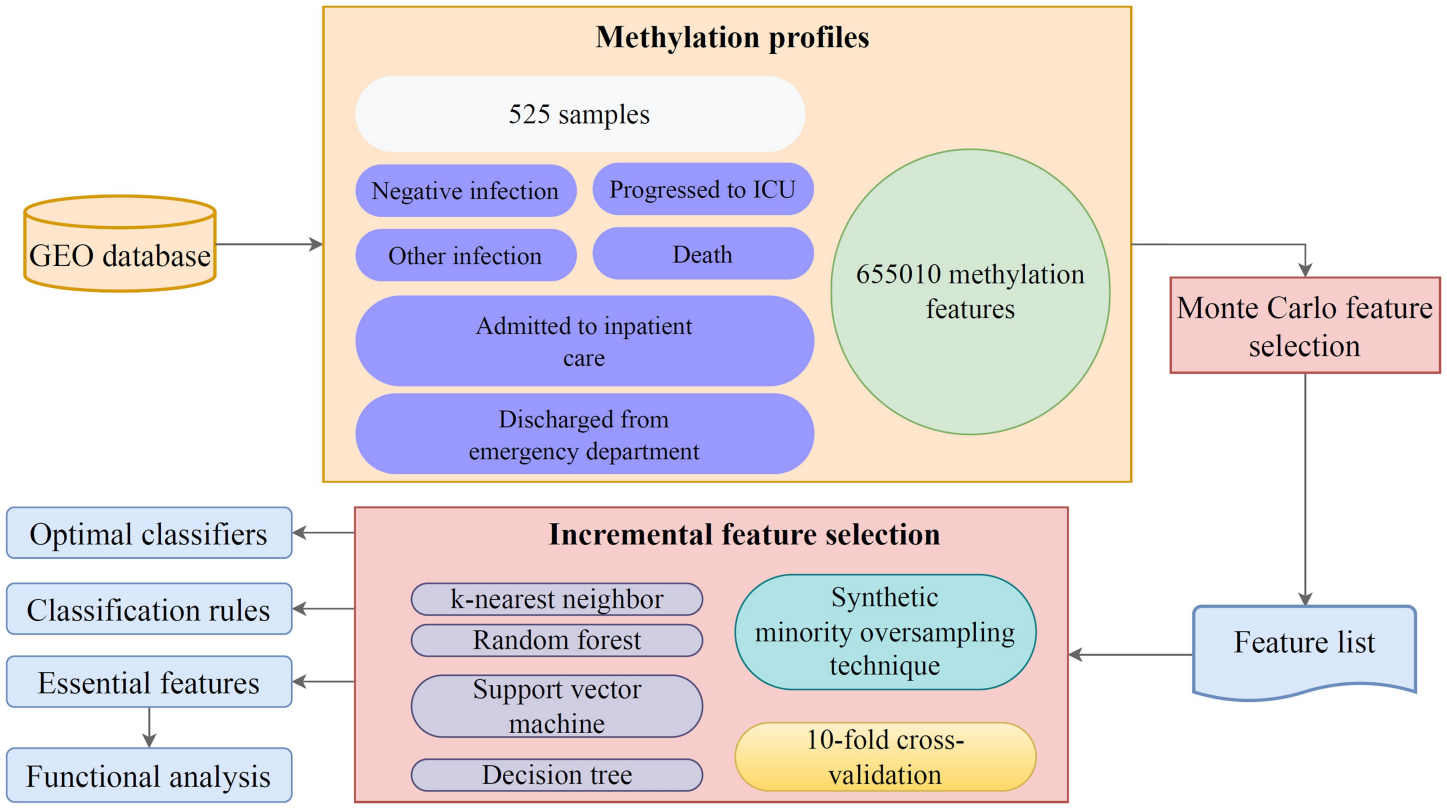
Workflow of this study. First, the Monte Carlo feature selection (MCFS) method was used to rank methylation signatures based on their importance, and a ranked feature list was generated. This list was then fed into the incremental feature selection (IFS) method with different classification algorithms to determine the optimal features for each classification algorithm. Optimal classifiers were set up. Classification rules generated by the optimal decision tree (DT) classifier were used to analyze the methylation expression pattern. The genes corresponding to essential methylation sites were subjected to functional enrichment analysis.

This study conducted a deep computational investigation on the blood methylation profile with six severity types from the GEO database. The entire procedures are illustrated in Figure 1. The MCFS method was first used to rank methylation features based on their importance, and a ranked feature list was generated. This list was then fed into the IFS method with different classification algorithms to determine the optimal features for each classification algorithm and construct optimal classifiers. Classification rules generated by the optimal DT classifier were used to analyze the expression pattern of key methylation features.

### Results of methylation feature ranking by the MCFS method

**Figure 2:**
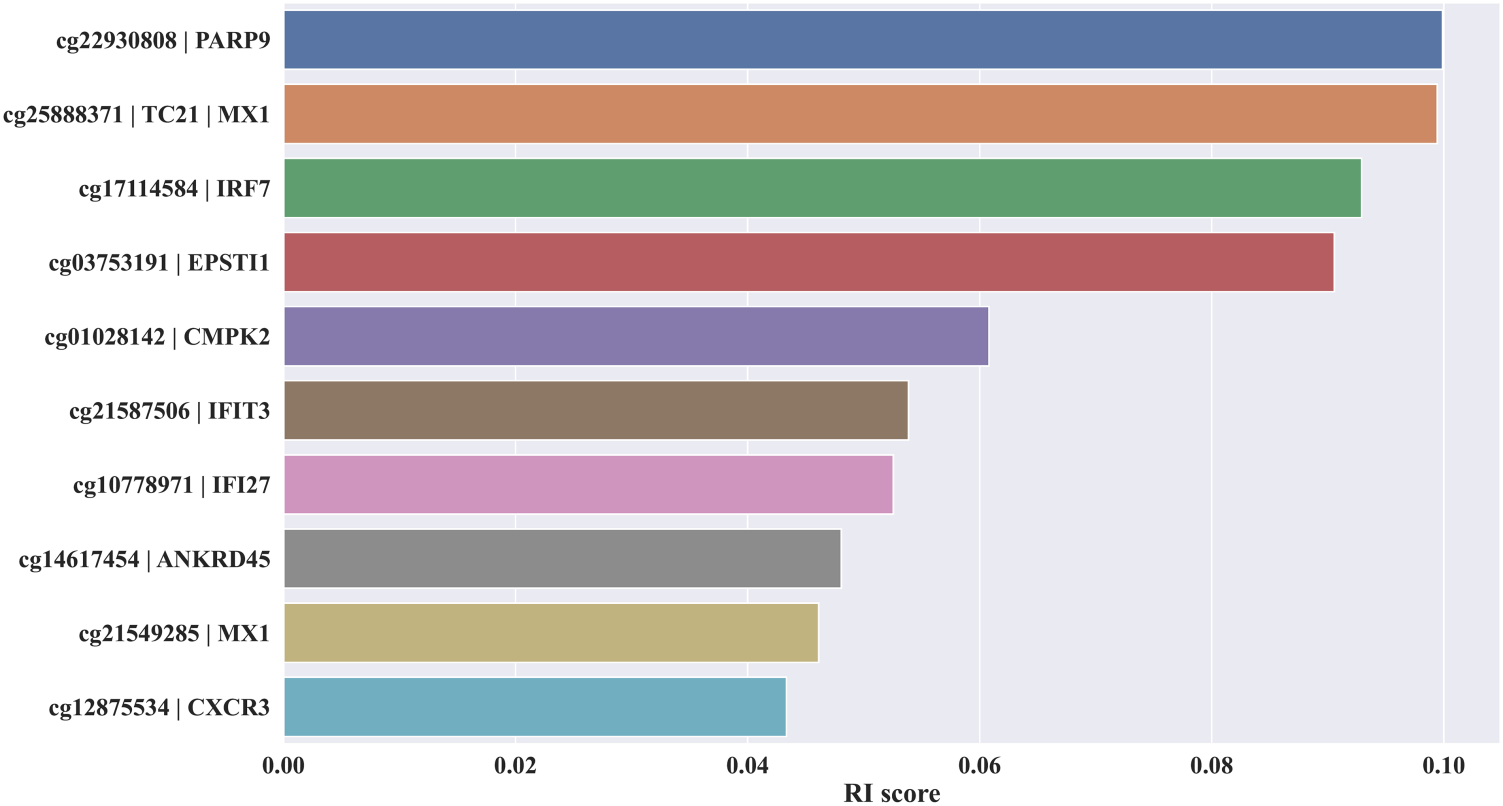
Bar chart to show top 10 key methylation features and their relative importance scores.

Initially, the MCFS method was used to rank 655,010 methylation features contained in blood the methylation profile. Each feature was assigned a RI score. A ranked feature list in descending order based on RI scores was generated. As some features were assigned RI scores of 0, they were removed. Thus, the final list contained 654,081 features with RI scores larger than 0, which is provided in Supplementary Table S1. The top 10 features alone with their RI score are plotted in Figure 2.

### Identification of the optimal number of methylation features with IFS

**Figure 3:**
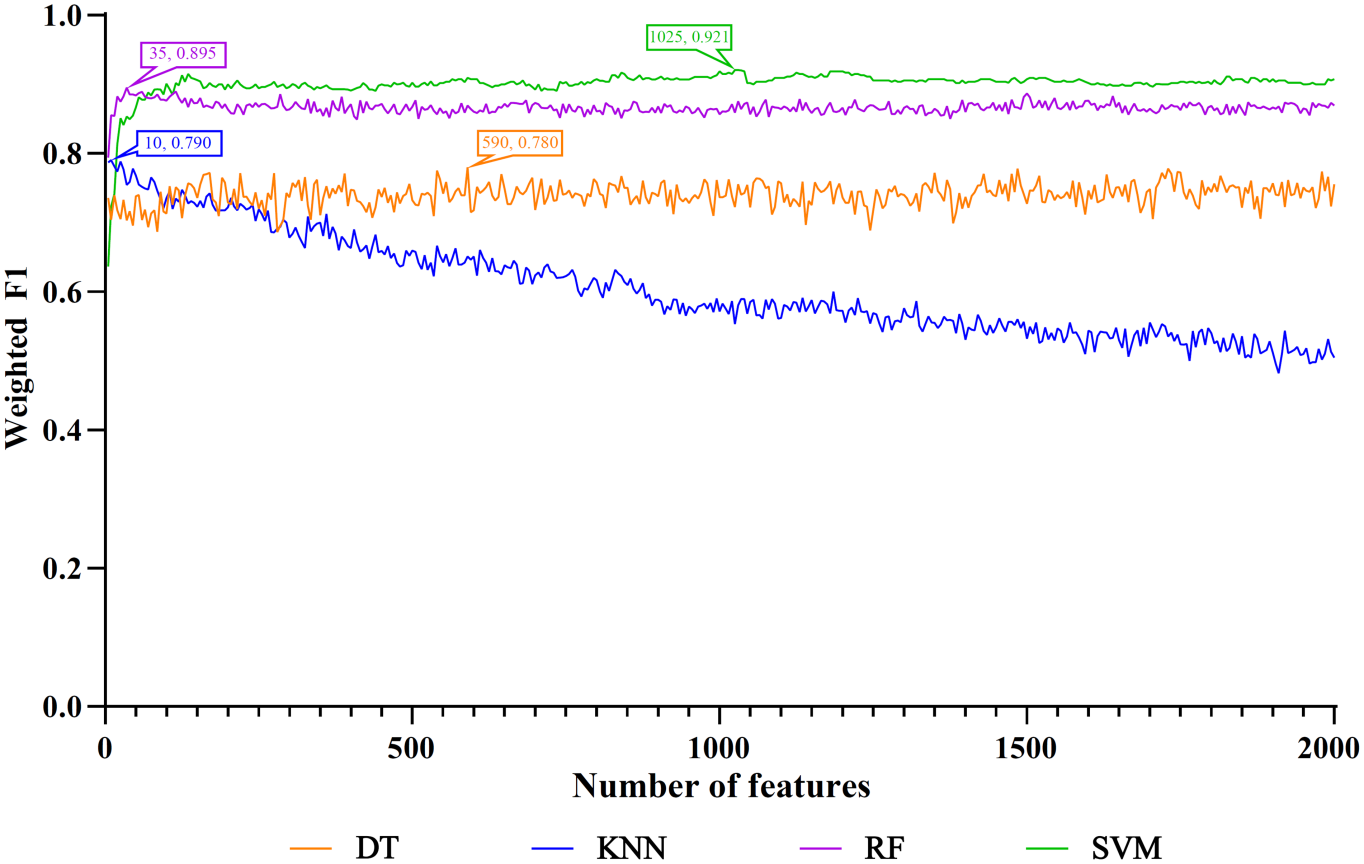
IFS curves to show the performance of different classification algorithms under different feature subsets. The highest weighted F1 for each classification algorithm was marked on the corresponding IFS curve. The SVM yielded the highest weighted F1 of 0.921 when top 1,025 features were used.

**Figure 4:**
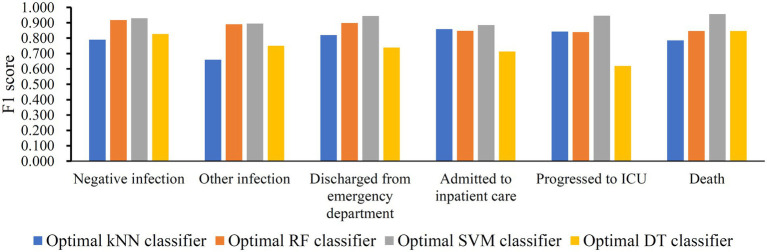
Performance of four optimal classifiers on six classes. The optimal SVM classifier produced best performance on all classes.

The IFS method was applied to determine the optimal features in the ranked feature list for each classification algorithm. To save time, we only considered top 2000 features in the list because of the huge number of features. The step size is set to five in the IFS method, thereby generating 400 feature subsets. The sample dataset comprising these feature subsets was learned by each of four classification algorithms, namely DT, kNN, RF, and SVM. Lots of classifiers were built, which were evaluated by 10-fold cross-validation. The evaluation metrics for each classifier are provided in Supplementary Table S2. To clearly display the performance of classifiers under different feature subsets, an IFS curve is plotted for each classification algorithm, which is provided in Figure 3. For SVM, the highest weighted F1 was 0.921 when top 1,025 features were adopted. These features constituted the optimal features for SVM and an optimal SVM classifier was built based on these features. As for kNN and RF, their highest weighted F1 values were 0.790 and 0.895, respectively. Their optimal features were top 10 and 35 features in the list. Furthermore, the optimal kNN and RF classifiers were set up with their optimal features, respectively. For DT, its highest weighted F1 was 0.780, which was obtained by using top 590 features. Such features comprised the optimal features for DT and the optimal DT classifier was built using these optimal features. According to the weighted F1 values of above optimal classifiers, the optimal SVM classifier was best, followed by the optimal RF and kNN classifiers, whereas the optimal DT classifier provided the lowest performance. Table 2 further lists other overall measurements for four optimal classifiers. It can be observed that on each measurement, the optimal SVM classifier always provided the highest performance, and the optimal RF classifier yielded slightly lower performance than the optimal SVM classifier. The performance of the other two optimal classifiers was much lower. The optimal DT classifier was a little inferior to the optimal kNN classifier. As for the performance of the above four optimal classifiers on six classes, it is illustrated in Figure 4. Clearly, the optimal SVM classifier generated the highest performance on all classes. On most classes, the optimal RF classifier occupied the second places. The optimal kNN and DT classifiers gave an almost equal performance. These results conformed to the overall performance of four optimal classifiers mentioned above.

**Table 2:** Overall performance of the optimal classifiers.

**Classification algorithm**	**Number of features**	**ACC**	**MCC**	**Macro F1**	**Weighted F1**
k-nearest neighbor	10	0.784	0.730	0.793	0.790
Random forest	35	0.893	0.842	0.873	0.895
Support vector machine	1,025	0.920	0.881	0.926	0.921
Decision tree	590	0.771	0.686	0.749	0.780

With the above arguments, the optimal SVM classifier was best. It can be an efficient tool to determine the severity of SARS-CoV-2 infection. The optimal RF classifier was inferior to the optimal SVM classifier. However, its efficiency was much higher than that of the optimal SVM classifier as much less features were used. This classifier can be used to conduct large-scale tests.

### Classification rules generated by the optimal DT classifier

**Figure 5:**
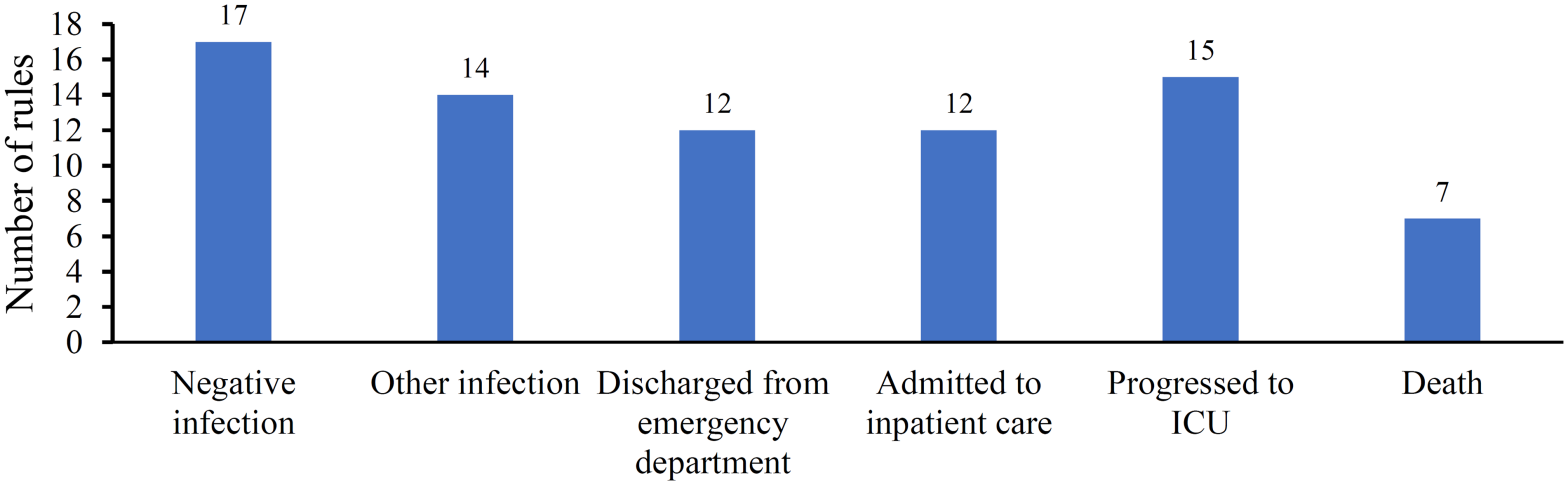
Distribution of classification rules on six classes.

Although the optimal DT classifier provided lower performance than the other three optimal classifiers, it can provide much more explicable information than other classifiers. As the optimal DT classifier adopted top 590 features in the list, a DT classifier trained with all samples comprising these features was built. Classification rules were extracted from the tree, resulting in 77 rules. These rules are provided in Supplementary Table S3. The number of rules for each class is displayed in Figure 5. The rules for “negative infection” were most, whereas those for “Death” were least. In section “Analysis of rules for different classes”, some rules would be discussed.

### Results of functional enrichment analysis

**Figure 6:**
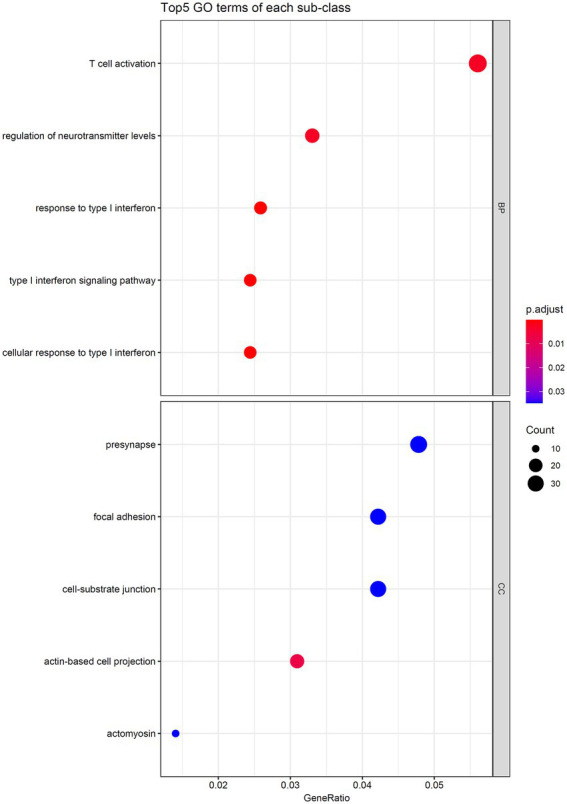
Top five GO terms enriched by the genes converted by the top 1,025 methylation features.

**Figure 7:**
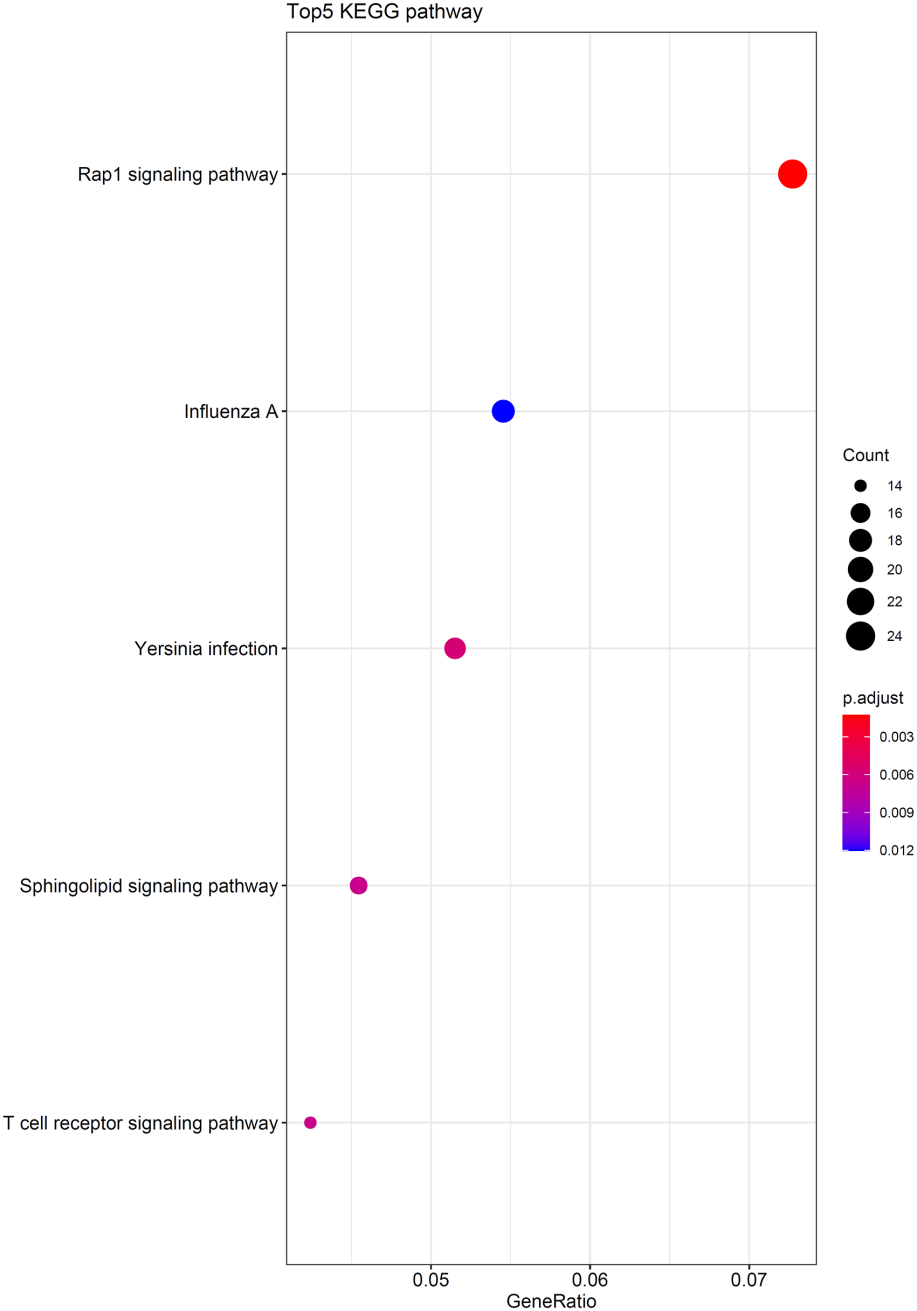
Top five KEGG pathways enriched by the genes converted by the top 1,025 methylation features.

As the optimal SVM classifier gave the best performance. This meant that features used in this classifier, that is the optimal features for SVM, were essential for determining the severity of SARS-CoV-2 infection. The corresponding genes of these features were picked up and the GO and KEGG enrichment analyses were performed on these genes, uncovering the biological meaning behind these genes. The detailed results are listed in Supplementary Table S4. Figures 6, 7 reveal that these genes were mainly enriched in biological processes, such as T-cell activation, regulation of neurotransmitter levels, and type I interferon signaling and KEGG pathways (e.g., Rap1 signaling pathway, Yersinia infection, and T-cell receptor signaling pathway). The role of these biological functions in SARS-CoV-2 infection will be verified in the section “Functional analysis based on GO and KEGG pathway”.

## Discussion

Most studies only distinguish COVID-19-positive and negative samples. In this study, based on blood methylation biomarkers, we can not only classify COVID-19 from negative controls and other infections, but also accurately predict the clinical outcome of COVID-positive patients in detail. In practice, the physicians can prioritize and allocate health and medical resources for COVID-19 patients based on their predicted severe clinical outcomes. For the least severe patient, they can be discharged from hospital and avoid medical resource overstretch. For the second least severe patient, they can be hospitalized but without intensive health care. For the severe patient, intensive health care should be prepared. For the most severe patient who may die from COVID-19, life support system should be prepared.

A variety of machine learning methods were used to investigate the methylation profile on severity of SARS-CoV-2 infection. Some essential methylation features that can characterize the severity of SARS-CoV-2 infection were identified. Furthermore, a set of rules were also set up, which can not only classify SARS-CoV-2 infection samples, but also depict the methylation patterns for different severity of SARS-CoV-2 infection. These methylation features and rules would then be discussed below.

### Analysis of essential features

Key methylation signatures that can be used to distinguish severity of SARS-CoV-2 infection were obtained by using a set of machine learning methods. The genes corresponding to the top-ranked methylation signatures, listed in Table 3, were analyzed to demonstrate the reliability of the results.

**Table 3:** Essential methylation sites and their corresponding genes for distinguishing severity of SARS-CoV-2 infection.

**Methylation sites**	**Gene symbol**	**Description**
cg22930808	PARP9	Poly (ADP-Ribose) Polymerase Family Member 9
cg25888371	MX1	MX Dynamin Like GTPase 1
cg17114584	IRF7	Interferon Regulatory Factor 7

As a type I IFN regulatory gene, high expressions of polyadenosine diphosphate ribose polymerase 9 (PARP9, cg22930808) accompanied by hypomethylation at relevant sites can enhance IFN signaling (Zhu et al., 2019), thereby playing a role in solid tumors, macrophage regulation, and antiviral immunity (Xing et al., 2021). PARP9 mediates the production of type I interferon after binding to viral RNA by activating the PI3K/AKT3 signaling pathway, thereby protecting against viral infection (Zhang et al., 2015). In addition, PARP9 is involved in the activation of anti-inflammatory M2 macrophages. This condition showed that the SARS-CoV-2 Nsp3 protein is similar to PARP9 and can inhibit PARP9 through molecular mimicry, depleting M2 macrophages, and weakening interferon signaling, which then weakens the ability of the host to resist viral infection (Da Silva et al., 2020; Fehr et al., 2020). The reduction of PARP9 combined with the reduction of NK and CD8+ cells leads to a weak viral response of the host, which may be an important reason for the life-threatening severe infections in patients.

Similar to PARP9, as an important host interferon-stimulated gene in antiviral infection (Anderson et al., 2021), MX1 (cg25888371) is hypomethylated in CpG after viral infection (Luo et al., 2021) and then participates in regulating the defense response of the host to infection. The study found that the expression of MX1 was significantly increased in COVID-19 patients compared with non-COVID-19 patients and increased with the viral load (Bizzotto et al., 2020). In addition, the methylation of CpG in MX1 is associated with the severity of HIV patients using cocaine in HIV infection studies (Shu et al., 2020), suggesting that MX1 methylation levels may be a reliable predictor of COVID-19 severity.

IRF7 (cg17114584), a member of the interferon regulatory factor (IRF) family, can regulate the response of type I IFN to viral infection. Phosphorylation of IRF7 upon pathogen stimulation followed by nuclear translocation induces the expression of IFN-α (Puthia et al., 2016). The methylation level of its promoter region affects the clinical manifestations of diseases (Konigsberg et al., 2021). Studies have shown that the expression level of IRF7 is increased in patients with mild/moderate COVID-19 (Li N. et al., 2021), while those with reduced IRF7 expression due to hypermethylation of the IRF7 promoter gene are likely to develop severe infection after SARS-CoV-2 infection (Liu and Hill, 2020).

Overall, the obtained genes showed differential expression of methylation in different infection groups, suggesting that the methylation status of different genes may be an important feature to distinguish different SARS-CoV2 infection severities.

### Analysis of rules for different classes

The decision rules (Supplementary Table S3) revealed the importance of IRF7 (cg17114584) in predicting the clinical outcome of SARS-CoV-2 infection. IRF7 is markedly hypermethylated in patients with poor clinical response (progressed to ICU or death) compared with patients with mild clinical response (discharged from the emergency department or admitted to inpatient care). This finding is consistent with a previous result, in which IRF7 can regulate the response of type I IFN to viral infection and the expression level is negatively correlated with clinical manifestations. Recent studies show that methylation levels of IRF7 correlate with COVID-19 severity (Barturen et al., 2021), which is also consistent with the conclusions in the data source literature (Konigsberg et al., 2021).

The decision rule for distinguishing between other infections and non-COVID-19/COVID-19 infections indicated that FHL1 (cg00012680) was highly methylated in patients with other infections. As a member of the FHL protein family, FHL1 is mainly expressed in the heart and skeletal muscles (Shathasivam et al., 2010). As a tumor suppressor gene, FHL1 is downregulated in a variety of tumors (Wang et al., 2017). Studies have also shown that FHL1 is associated with viral infections (e.g., acting as a host factor to promote chikungunya virus infection; Meertens et al., 2019). Conversely, patients in the “death” cohort had low levels of FHL1 methylation. A study has shown that in COVID-19 patients, FHL1 is associated with the JAK–STAT pathway, which can indirectly activate STATs and induce various inflammatory responses (Bass et al., 2021). Another key criterion in distinguishing patients from other infections is the methylation level of TGFB3 (cg06958766), which is hypomethylated in COVID-19 patients (especially ICU and death patients). Existing studies have demonstrated that TGFB3 is a gene related to immune dysregulation in cardiovascular disease, and its expression is also dysregulated in COVID-19 (Lee et al., 2021). The association of the methylation level of TGFB3 with the clinical outcome of COVID-19 infection has not been revealed, and such level is speculated to be possibly associated with poor clinical response to COVID-19.

The result also indicated that the methylation level of the interferon type I pathway-related gene RSAD2 (cg10549986) for COVID-19 patients was negatively correlated to the severity of COVID-19, and the expression of RSAD2 is reported to have reached the highest level in the early stage compared with the late stage of COVID-19 (Zhang C. et al., 2021). This finding may be related to the decrease in IFN activity in patients with severe infections. In COVID-19 patients, RSAD2 can enhance antiviral and immunomodulatory functions after viral infection, and patients discharged from the emergency department in the current results had lower levels of RSAD2 methylation, possibly related to high RSAD2 expression levels and enhanced antiviral immunity (Zhu et al., 2020).

### Functional analysis based on go and KEGG pathway

T-cell activation is the most significantly enriched pathway. Studies have shown that RNA m6A methylation is crucial for controlling the activation and differentiation of T lymphocytes (Qiu et al., 2021). m6A with T-cell activation function mainly mediates the activation and proliferation of T cells by increasing TGF-β and PI3K-AKT signaling necessary for T-cell differentiation and plays an anti-COVID-19 role (Li et al., 2017). Increased m6A regulator expression in COVID-19 patients results in the high expression of activated CD4 memory T cells (Yao et al., 2021). As a crucial immune cell in SARS-CoV-2 infection, T cells have dual roles in patients with COVID-19. The expression level of T cells is increased in patients with mild infection; among which, CD8^+^ T cells highly express cytotoxic molecules, such as granzyme A, which play an antiviral immune effect (Liao et al., 2020). Meanwhile, the expression levels of cytotoxic molecules and Tregs in severe patients are reduced (De Biasi et al., 2020; Toor et al., 2021). Studies have shown the presence of a complete memory T-cell response in asymptomatic or mildly infected COVID-19 patients (Sekine et al., 2020) and detected SARS-CoV-2-related T-cell responses in healthy blood samples, which may be due to seasonal coronavirus-induced T-cell responses and may further prevent serious infections (Braun et al., 2020; Mateus et al., 2020).

The current study also observed enrichment of pathways that regulate the level of neurotransmitters, suggesting the role of methylation of neurotransmitter-related genes in immunity to virus infection. Studies have shown that in addition to macrophages, viral infection also activates mast cells to release histamine, arachidonic acid, and other neurotransmitters, and histamine can strongly raise the level of IL-1, which, in turn, increases lung inflammation in SARS-CoV-2 infection (Conti et al., 2020). Furthermore, SARS-CoV2 infection will reduce the synthesis of dopamine and acetylcholine, resulting in the weakened immune function of the body (Blum et al., 2020; Alexandris et al., 2021).

Type I interferon plays a crucial role in antiviral immunity, and studies have shown that hypermethylation of IFN-related genes is a unique methylation signature of severe COVID-19. Moreover, three of the significant enrichment pathways are related to type I interferon response, further confirming the important role of IFN-related methylation in determining the severity of COVID-19 and the reliability of the current study. *In vivo*, IFN can bind to IFN receptors in an autocrine and paracrine manner to activate the JAK/STAT signaling pathway, thus demonstrating antiviral effects (Liu et al., 2012). IFN activity was also lower in patients with severe infection than mild infection patients, and impaired IFN-α production is an important sign of severe infection (Hadjadj et al., 2020), which may be related to the hypermethylation of IFN-related genes and the inhibition of the expression of related genes. In addition, studies have shown that IFN expression is delayed in SARS-CoV-2 infection (Kim et al., 2016). Such a delay leads to high levels of interferon expression in severely infected patients but does not reduce viral load; meanwhile, IFN pretreatment can significantly reduce viral infection levels, suggesting that drugs that can boost IFN production may be an effective option for early treatment of SARS-CoV-2 (Park and Iwasaki, 2020).

The enrichment of cellular components of differentially methylated genes mainly focused on the virus infection process of cells, including cell junction and migration. Synapses mainly mediate information transmission between neurons; they can also transmit large particles and mediate virus particles into the central nervous system, thus reflecting the neuroinvasiveness of coronaviruses (Li et al., 2020). As the main site of cell adhesion, focal adhesions help viral particles enter cells, and its functional integrity is critical to the infection and spread of SARS-CoV-2 (Sulzmaier et al., 2014).

This study also found enrichment of differentially methylated genes in the RAP1 pathway. RAP1 pathway plays an important role in processes, such as cell adhesion, junction, and polarity, and promotes tumor cell invasion and migration (Looi et al., 2020). Pulmonary vascular barrier integrity defection is a fatal factor in severe COVID-19 patients (Yamamoto et al., 2021). Meanwhile, studies have found that RAP1 can enhance endothelial cell–cell junctions mediated by VE-cadherin and regulate vascular permeability (Rho et al., 2017), suggesting that the RAP1 signaling pathway may serve as a potential therapeutic target for COVID-19.

The analysis of key features and related decision rules verified the effectiveness of methylation status in distinguishing different states of SARS-CoV-2 infection, which will provide a reference for studying the stratification of patients and help develop new treatment strategies.

## Conclusion

A computational workflow containing several machine learning methods was designed to identify the blood methylation features and their expression rules, which can distinguish the severity of SARS-CoV-2 infection. First, the methylation features in the expression profile were analyzed by the MCFS algorithm, producing a ranked feature list. Next, this list was introduced into the IFS method to generate a series of feature subsets. Different classification algorithms were used to train samples comprising these feature subsets to build classifiers. After evaluating their performance, the optimal features were determined. The classification rules were extracted by the optimal DT classifier. The essential features were analyzed by functional enrichment to detect their biofunctional information. Some key features and rules are justified by recently published academic literature, which provides a reference for further related research.

## Data availability statement

Publicly available datasets were analyzed in this study. These data can be found at: https://www.ncbi.nlm.nih.gov/geo/query/acc.cgi?acc=GSE167202.

## Author contributions

TH and Y-DC designed the study. SD and KF performed the experiments. ZL, MM, and XZ analyzed the results. ZL, MM, and SD wrote the manuscript. All authors contributed to the article and approved the submitted version.

## Funding

This work was supported by the Strategic Priority Research Program of Chinese Academy of Sciences (XDA26040304 and XDB38050200), National Key R&D Program of China (2018YFC0910403), and the Fund of the Key Laboratory of Tissue Microenvironment and Tumor of Chinese Academy of Sciences (202002).

## Conflict of interest

The authors declare that the research was conducted in the absence of any commercial or financial relationships that could be construed as a potential conflict of interest.

## Publisher’s note

All claims expressed in this article are solely those of the authors and do not necessarily represent those of their affiliated organizations, or those of the publisher, the editors and the reviewers. Any product that may be evaluated in this article, or claim that may be made by its manufacturer, is not guaranteed or endorsed by the publisher.

## Footnotes

^1^ https://imbalanced-learn.org/stable/
